# Genetic diversity of *Actinidia* spp. shapes the oomycete pattern associated with Kiwifruit Vine Decline Syndrome (KVDS)

**DOI:** 10.1038/s41598-023-43754-y

**Published:** 2023-09-30

**Authors:** Giovanni Mian, Guido Cipriani, Giuseppe Firrao, Marta Martini, Paolo Ermacora

**Affiliations:** Department of Agricultural, Food, Environmental and Animal Sciences, University of Udine, Via delle Scienze 206, 33100 Udine, Italy

**Keywords:** Pathogens, Biotic, Flooding

## Abstract

Kiwifruit Vine Decline Syndrome (KVDS) is an important soil-borne disease for the Italian kiwifruit industry, causing €300,000 in economic losses in 2020 alone. So far, the organisms recognized as involved in the aetiology of KVDS mainly belong to the *Oomycota*. As no effective management strategies exist, a promising approach to overcoming KVDS is the use of resistant species as rootstocks or for inclusion in breeding programs. Several *Actinidia* genotypes showing different level of resistance to KVDS were grown in disease-promoting soils. A metabarcoding approach was set up to identify KVDS-associated oomycetes and investigate whether the main species involved may vary according to plant genotype. Our results clearly showed significant differences between the genotypes in terms of oomycetes present in both plant rhizosphere and endosphere, which were strongly correlated with the symptoms displayed. We found out that the resistance of *Actinidia macrosperma* to KVDS is related to its ability to shape the pathobiome, particularly as far as the endosphere is concerned. In our conditions, *Phytophthora* sp. was predominantly found in sensitive genotypes, whilst *Globisporangium intermedium* was mainly detected in asymptomatic plants, suggesting that the latter species could compete with the recruitment of *Phytophthora* sp. in plants with different levels of resistance, consequently, explaining the onset of symptoms and the resistance condition.

## Introduction

Since 2013, *Actinidia* cultivation has been affected by a disease named Kiwifruit Vine Decline Syndrome (KDVS)^1^, which has led to significant economic losses reaching 300 M€ in 2020 in Italy^2^. In KVDS symptomatic plants, the root system is heavily compromised due to the presence of extensive rot on structural roots and the absence of feeding roots. Consequently, the aerial part collapses with the first heat waves^1^. The involvement of many soil-borne pathogens as KVDS aetiological agents has been ascertained, among which the oomycetes seem to be the most important. *Phytophthora* (*Phy.*) spp., *Pythium (Py.)* spp., and *Phytopythium (Pp.)* spp. have frequently been isolated from infected plants^1,3,4^. Some isolates of *Phy. cryptogea*, *Phy. citrophthora, Pp. vexans* and *Pp. chamaehyphon* have also been demonstrated to be pathogenic on kiwifruit cuttings^5,6^. Furthermore, the activity of soil-borne pathogens is enhanced by abiotic conditions, among which the role of waterlogging is fundamental^1^, indirectly confirming the importance of the oomycetes as their life cycle is strictly related to water^7,8^.

Plants have multiple compartments (e.g., root endosphere and rhizosphere, etc.), each of which offers unique habitats for microbes and differs in many aspects that differentially regulate them (e.g., structural, microenvironmental features, etc.)^9–11^. *Actinidia* roots, as well as in other plants, are heavily colonized by complex communities, consisting of bacteria, fungi, oomycetes, etc. (the so-called root microbiome)^1,12^. Furthermore, host plants and their soil-borne pathogens form complex pathosystems^13^, as plants have evolved traits that govern root microbial assemblages ^14,15^. The rhizosphere is the narrow soil zone directly surrounding the root system, highly modulated by roots^16^. The microbial community of the rhizosphere varies among different plant genotypes^17^, and here plants can positively or negatively select for community members that can promote plant growth^18,19^. On this basis, the genetics of the host can shape the plant-pathobiome structure; in fact, Escudero-Martinez and co-workers^20^ found that the plant endosphere has the potential to attract or filter the microbes inhabiting the rhizosphere. Thus, the aforementioned observations indicate that microbes are selectively chosen from within the root in response to multiple signals from the plant^21–23^, where in the context of KVDS, the pathogens follow the same trend, especially for the most studied (i.e., oomycetes)^24–27^.

Nevertheless, little is known about the relationship between the soil-borne oomycete pattern and the different behaviour of the *Actinidia* genotypes (resistant or sensitive) as well as how the host can influence their composition. So far, only Yano and co-workers^28^ found *Actinidia* species tolerant to *Pp. helicoides* and *Pp*. *vexans* causing root rot (hence, a form of KVDS), while Mian et al.^29^ reported on-field resistance of *A. macrosperma*, *A. arguta* and the rootstock Bounty71 grown in KVDS-promoting soils. However, evidences in other species suggest a difference in the pathosystem composition between different genotypes^30,31^.

Metabarcoding (i.e., targeting a specific genomic barcode conserved among kingdoms) provides a comprehensive picture of the genetic diversity present in a sample^32^, thus, it is a valuable tool for identifying putative causal agents in the context of complex diseases like KVDS^4,33^. We have previously reported the potential of different *Actinidia* species to survive and grow in KVDS promoting soils, since it is of great interest to have different phenotypically resistant genotypes to be used as parents in a rootstock breeding projects^34^. Hence, the main objectives of our work were to assess, describe and compare the structure and composition of the oomycete pattern in root rhizosphere and endosphere of five *Actinidia* genotypes with different level of resistance to KVDS, applying a metabarcoding approach.

## Results

### Assessment of KVDS root symptoms and root volume

**Figure 1:**
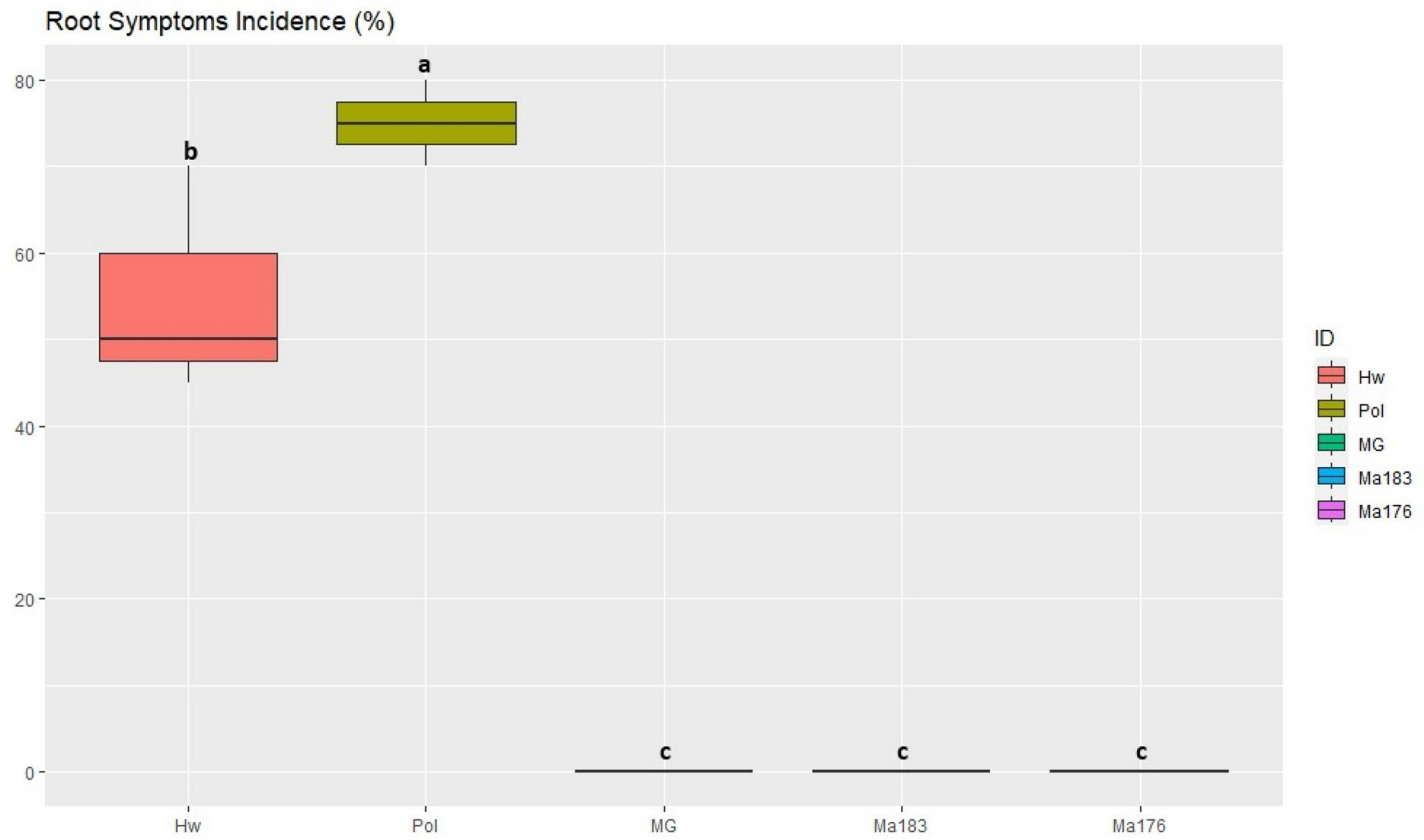
Incidence of root symptoms (percentage of root necrosis). Data are the mean of 8 replicates per genotype. Statistical analysis (ANOVA) was carried out using the Tukey HSD test using R version 4.3.0. Values assigned with different letters are significantly different at *α* < 0.05. Hw: *A. deliciosa* cv. Hayward, Pol: *A. polygama*, MG: *A. arguta* cv. Miss Green, Ma183 and Ma176: *A. macrosperma.*

The characteristic symptoms of KVDS, including root necrosis and rat tail symptoms, were observed upon the uprooting of plants, concomitant with maximum canopy decay. Radical system of *A. macrosperma* accession 176 (Ma176), *A. macrosperma* accession 183 (Ma183), and *A. arguta* cv. Miss Green (MG) exhibited no typical disease symptoms. In contrast, *A. polygama* (Pol) and *A. chinensis* var. *deliciosa* cv. Hayward (Hw) displayed severe affliction, characterized by extensive root compromise and an average root necrosis level ranging from 60 to 70% (Fig. 1). These findings corroborate prior observations made in an open-field environment study^34^. Further details are elucidated in Figure S2, providing a visual representation of symptom severity.

In terms of root volume, when we transplanted the plants into pots containing soil that promotes KVDS, we found that the root system volumes (mL) were quite similar among the various species, although specific data is not presented here. However, when we uprooted the plants (Figure S3), we observed that the root volumes for Ma176, Ma183, and MG were the highest, measuring 160 mL, 200 mL, and 205 mL, respectively. Importantly, these differences between these genotypes were not statistically significant, but they were notably larger compared to Pol (75 mL) and Hw (35 mL). Additionally, Pol exhibited a significantly larger root volume compared to Hw.

Furthermore, we assessed the canopy dry weight after the vegetative season (as detailed in Figure S7). Among the species, Ma176 had the highest canopy dry weight, measuring 323 g, which was significantly greater than Ma183 and MG, which weighed 160 g and 170 g, respectively. In contrast, Pol had a dry weight of 48 g, while Hw had the lowest, at 30 g.

### Quality metrics of sequencing

A total of 7,435,581 reads were recovered from Illumina MiSeq sequencing. The sequences were evenly distributed among the samples. After removing contaminants, trimming adapters, filtering quality, and deleting chimeric reads, about 40% of the sequences were removed, and an average of about 40,000 reads per sample were retained for analysis. None of the reads were classified as plant DNA. After filtering, approximately 2,974,232 reads remained as singletons. All rarefaction curves reached a saturation plateau; thus, all samples were suitable for further analysis, with optimal good coverage (indicator: from 0.7 to 1—data not shown).

### Alfa diversity of the oomycetes in the rhizosphere and endosphere of kiwifruit plants

**Figure 2:**
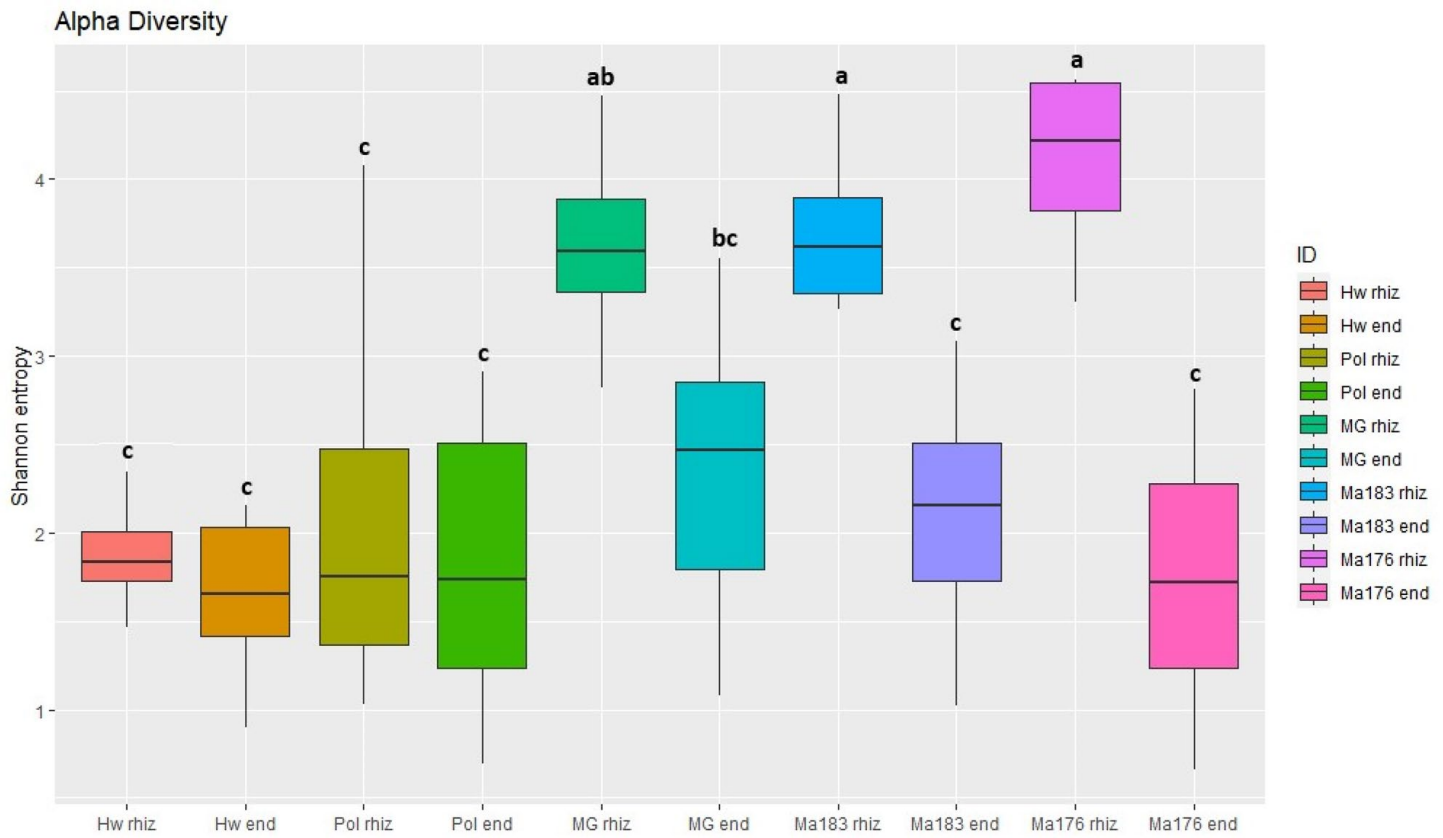
Statistical analysis of oomycetes alpha-diversity by root rhizosphere and endosphere considering Shannon’s H index (entropy). Significant effects were tested using a pairwise Kruskal–Wallis H test. Values assigned by different letters are significantly different at α < 0.05. Data are the mean of 8 replicates per genotype. Hw: *A. deliciosa* cv. Hayward, Pol: *A. polygama*, MG: *A. arguta* cv. Miss Green, Ma183 and Ma176: *A. macrosperma*. rhiz: rhizosphere, end: endosphere.

The results of the alpha-diversity indices (Shannon entropy) were highly dependent on the compartment considered. A great significant diversity was observed in the rhizosphere (rhiz) of Ma176, Ma183, and MG compared to Hw and Pol, which showed no significant differences between them. Concerning the root endosphere (end), no significant differences emerged between the genotypes tested. The differences among each genotype, including rhiz and end compartments, were statistically more pronounced for Ma176 rhiz, Ma183 rhiz, and MG rhiz compared to all other genotype compartments. Among these four, Ma176 rhiz and Ma183 rhiz exhibited similar levels, both slightly higher than MG rhiz, which in turn was slightly elevated compared to MG end (Fig. 2). As expected, the Shannon entropy was significantly higher in the rhizosphere rather than in the root endosphere, regardless of genotype (Figure S4).

### Oomycete patterns in the rhizosphere and endosphere compartments

We limited the analysis to the 185 most frequent features, i.e., those that received at least 0.01% of the total hits. ASVs (Amplicon Sequence Variants) were grouped into 51 OTUs (Operation Taxonomic Units) assigned to the phylogenetically defined clusters that resulted from the large study of Robideau and collaborators^35^. Compressively, only 7 OTUs accounted for more than 90% of the total hits assigned to oomycetes. In particular, three OTUs were prominent, as they built up to 50–80% of the total hits in each sample, although their relative proportion varied greatly depending on the compartment (endosphere-rhizosphere) and the host genotype, as discussed in the next paragraphs. The first and the third most frequent OTUs among the samples were members of the *Pythium* clade F, a group recently reclassified in the genus *Globisporangium*. These two OTUs, that were 98% identical in of the DNA sequences among themselves, clustered with the related species *G. intermedium* and *G. attrantheridium*, respectively, which have been shown to form a species complex^36^, clearly separated from other *Globisporangium* species. The other prominent OTU in the data set, which received nearly one third of the total hits and was the second most frequent OTU, clustered with the *Phytophthora* sp. (clade 7) according to the 100% identity of the DNA sequence with several entries of the dataset of Robideau and collaborators^35^, namely sequences obtained from strains of the species *Phy. cajani*, *Phy. cinnamomi*, *Phy. melonis*, *Phy. sinensis*, *Phy. sojae*, *Phy*. *niederhauserii*, and other species which are retained within clades 7b and 7c in the expanded phylogeny proposed by Yang and collaborators^37^. These clades include oomycetes that are regarded as major, destructive pathogens, such as *Phytophthora sojae*. Most of the other oomycetes highly frequent in our sampling were *Pythiaceae* belonging to the genus *Phytopythium* or to *Pythium* clades A, B, E and J.

Concerning the taxonomical identification, as a first step, we analysed the metabarcoding data of the oomycete pattern before transplantation, i.e., at the beginning of the experiments. It should be noted that the oomycete patterns in both rhizosphere and endosphere were completely different compared to what was found at the moment of maximum expression of symptoms after transplantation. The results are summarized in Figure S5.

**Figure 3:**
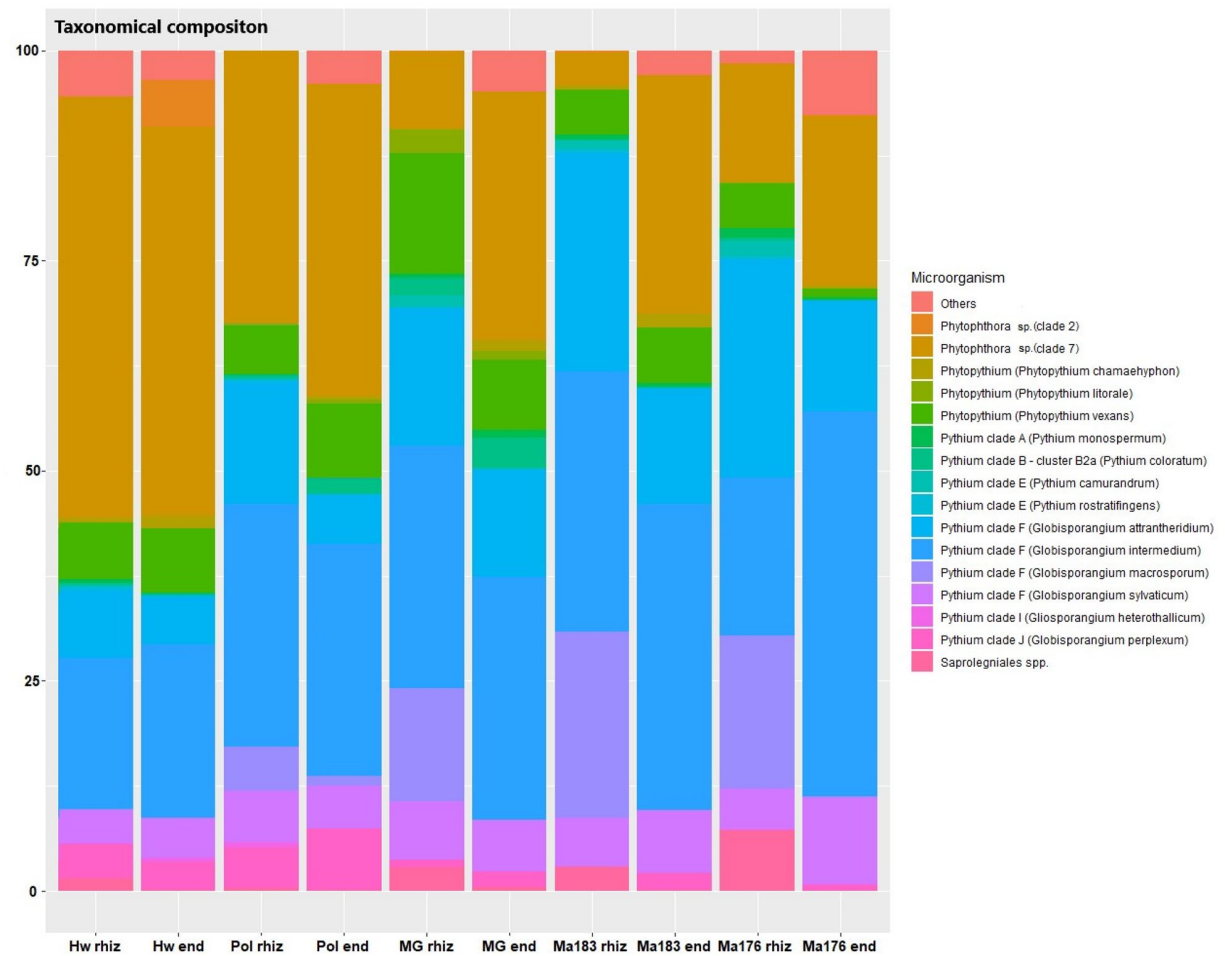
Taxonomic composition of the oomycetes found in the rhizosphere and endosphere of different *Actinidia* spp. at the time of full symptom development. Hw: *A. deliciosa* cv. Hayward, Pol: *A. polygama*, MG: *A. arguta* cv. Miss Green, Ma183 and Ma176: *A. macrosperma*. rhiz: rhizosphere, end: endosphere. Data are expressed as relative abundance (%) given by the number of reads for each microorganism on the total number of reads.

Regarding the oomycete patterns characterized at the moment of maximum expression of symptoms, the results will be discussed hereafter for both compartments in each *Actinidia* species under investigation. Differences among the evaluated genotypes for each OTU were estimated by applying a one-way ANOVA. Furthermore, in each replicate/plant, we found a similar number of oomycete sequences. Four out of the 15 OTUs identified as oomycetes represented 77% of the total reads obtained. These were the OTUs identified as *Phytophthora* sp. (clade 7) and three OTUs identified as members of *Pythium* clade F, namely *G. intermedium*, *G. attrantheridium*, and *G. macrosporum*. The results indicate that *Phytophthora* sp. (clade 7) is more prevalent in the sensitive genotypes (Hw and Pol) as compared to the resistant ones (Ma176, Ma183 and MG). Conversely, *Globisporangium* spp. are more prevalent in the resistant genotypes. This difference is particularly evident in the rhizosphere compartment, suggesting that the sensitive genotypes could be strongly colonized by pathogenic *Phytophthora* sp. (clade 7). The divergence is less significant in the endosphere compartment, which may indicate a less significant difference in restricting pathogen growth within the host plant tissue. It is also noteworthy that in the samples where *Phytophthora* sp. (clade 7) is not the predominant oomycete, the composition is mainly comprised of different *Globisporangium* species, with a consistent pattern within the replicates. The results also revealed the presence of *Pp. vexans* and *Pp. chamaehyphon* species which have been reported as relevantly involved in the KVSD on cv. Hayward^5,6^ (Fig. 3 and Table 1).

**Table 1:** One-way analysis of variance (ANOVA) of the oomycete taxonomic composition of the root rhizosphere and endosphere. Data are the percentage of reads recorded in each sample (mean of 8 replicates per genotype) on the total number of reads assigned to the Oomycota. Within rows, values designated by different letters are significantly different at α < 0.05. Statistical analysis was carried out using the Tukey HSD test *via* R version 4.3.0. Hw: *A. deliciosa* cv. Hayward, Pol: *A.polygama*, MG: *A. arguta* cv. Miss Green, Ma183 and Ma176: *A. macrosperma*.

	Hw	Pol	MG	Ma183	Ma176
Genotype rhizosphere					
*Pythium* clade F (*Globisporangium intermedium*)	18.041 b	28.97 a	28.792 a	31.022 a	18.861 b
*Phytophthora* sp. (clade 7)	50.125 a	32.39 b	9.196 c	3.958 d	14.021 c
*Pythium* clade F (*Globisporangium attrantheridium*)	8.063 c	14.60 b	16.389 b	26.040 a	25.973 a
*Phytopythium* (*Phytopythium vexans*)	6.734 b	5.81 b	14.352 a	5.305 b	5.258 b
*Pythium* clade F (*Globisporangium sylvaticum*)	3.936 a	6.12 a	7.034 a	5.741 a	4.821 a
*Pythium* clade J (*Globisporangium perplexum*)	4.042 a	4.860 a	0.852 b	0.075 b	0.115 b
*Pythium* clade B—cluster B2a (*Pythium coloratum*)	0.302 b	0.389 b	2.069 a	0.121 b	0.356 b
*Phytopythium* (*Phytopythium chamaehyphon*)	0.555 a	0.237 a	0.097 b	0.498 a	0.306 a
*Phytophthora* sp. (clade 2)	0.007 b	0.011 b	0.076 b	0.188 a	0.024 b
*Pythium* clade A (*Pythium monospermum*)	0.416 b	0.142 b	0.532 b	0.655 b	1.209 a
*Phytopythium* (*Phytopythium litorale*)	0.000 c	0.052 b	2.829 a	0.000 c	0.002 c
*Pythium* clade E (*Pythium camurandrum*)	0.198 b	0.185 b	1.381 a	0.982 a	1.840 a
*Pythium* clade F (*Globisporangium macrosporum*)	0.090 c	5.216 b	13.492 a	22.139 a	18.210 a
*Pythium* clade I (*Globisporangium heterothallicum*)	0.106 a	0.58 a	0.000 b	0.000 b	0.000 b
*Pythium* clade E (*Pythium rostratifingens*)	0.387 a	0.0808 a	0.119 a	0.425 a	0.332a
*Saprolegniales* spp.	1.536 c	0 d	1.182 c	2.841 b	6.955 a
Others	5.461 a	0.000 c	0.000 c	0.005 c	1.364 b
Genotype endosphere					
*Pythium* clade F (*Globisporangium intermedium*)	20.670 b	27.560 b	28.903 b	36.380 a	45.772 a
*Phytophthora* sp. (clade 7)	46.194 a	37.134 a	29.574 b	28.467 b	20.299 b
*Pythium* clade F (*Globisporangium attrantheridium*)	5.888 b	5.911 b	12.681 a	13.848 a	13.249 a
*Phytopythium* (*Phytopythium vexans*)	7.688 a	8.779 a	8.380 a	6.606 a	1.136 b
*Pythium* clade F (*Globisporangium sylvaticum*)	4.664 a	5.178 a	6.140 a	7.504 a	10.400 a
*Pythium* clade J (*Globisporangium perplexum*)	3.416 a	7.249 a	1.749 b	1.912 b	0.641 b
*Pythium* clade B—cluster B2a (*Pythium coloratum*)	0.164 b	1.811 a	3.676 a	0.096 b	0.020 b
*Phytopythium* (*Phytopythium chamaehyphon*)	1.514 a	0.394 b	1.291 a	1.532 a	0.241 b
*Phytophthora* sp. (clade 2)	5.610 a	0.000 b	0.000 b	0.003 b	0.058 b
*Pythium* clade A (*Pythium monospermum*)	0.134 a	0.204 a	0.852 a	0.454 a	0.285 a
*Phytopythium* (*Phytopythium litorale*)	0.004 b	0.533 a	1.034a	0.025 b	0.000 b
*Pythium* clade E (*Pythium camurandrum*)	0.000 b	0.008 b	0.147 a	0.003 b	0.000 b
*Pythium* clade F (*Globisporangium macrosporum*)	0.000 b	1.110 a	0.000 b	0.000 b	0.127 b
*Pythium* clade I (*Globisporangium heterothallicum*)	0.493 a	0.018 a	0.094 a	0.121 a	0.103 a
*Pythium* clade E (*Pythium rostratifingens*)	0.006 a	0.000 a	0.100 a	0.000 a	0.000 a
*Saprolegniales* spp.	0.054 c	0.126 b	0.500 a	0.128 b	0 c
Others	3.500 a	3.984 a	4.882 a	2.919 a	7.671 a

The Pearson correlation test shows that *Pythium* clade F (*Globisporangium intermedium*) is negatively correlated with symptoms (r = − 0.40), and it is also negatively correlated with the presence of *Phytophthora* sp. (clade 7) (r = − 0.34). Hence, the more *Pythium* clade F (*Globisporangium intermedium*) is present, the fewer symptoms and lower presence of *Phytophthora* sp. (clade 7) occur. Conversely, the more *Phytophthora* sp. (clade 7) is present, the more severity of symptoms develops, according to a positive correlation between *Phytophthora* sp. (clade 7) and symptoms (r = 0.44) (Figure S6).

## Discussion

Despite its recent appearance, KVDS is widely distributed in Italy and causes severe economic loss. It is associated to a dysbiosis at root level caused by a combination of biotic and abiotic factors. Plant health is strictly related to a complex microbiome which may include also pathogenic microbes, the pathobiome, whose ability to damage the plant is influenced by various mechanisms^38–40^. In this sense, we decided to focus on the *Oomycota* (also referred as water moulds) due to their life cycle strictly related to gravitational water and the high incidence of KVDS that has been reported in several orchards characterized by periods of waterlogging conditions^5,6^. Since the role of the *Actinidia* host plant in shaping the oomycete pattern for KVDS is currently unknown, with this study we introduce metabarcoding analysis to describe the microbial diversity in the root rhizosphere and endosphere of different *Actinidia* species in relation to KVDS.

In detail, the lower complexity observed in samples from the root endosphere compared to those from the rhizosphere is in agreement with what has been observed in similar studies for other pathosystems^41^. This is an important first point, as resistant genotypes may have the potential to exclude the pathogen and/or to recruit more beneficial microorganisms/less pathogenic ones than susceptible genotypes^33,42,43^, of course with a different capability and thus with a different level of resistance.

Regarding the oomycete pattern, the results reported in the literature for other holobiomes (i.e., not related to *Actinidia*) are not consistent; some studies have reported a clear separation of the communities of the two compartments, while others a partial or total overlap^44,45^. In our study, the oomycete pattern, both in the root endosphere and in the rhizosphere, resulted rather simple, being characterized by the presence of few dominant species. In both the rhizosphere and the endosphere, *Pythium* clade F (*Globisporangium* spp.), on one hand, and *Phytophthora* sp. (clade 7), on the other, were dominant in KVDS resistant genotypes (Ma176, Ma183, and MG) and in KVDS susceptible genotypes (Hw and Pol), respectively. Similar findings were reported by studies on holm oak decline, where in the presence of a high occurrence of *Phytophthora* spp., the abundance of other oomycetes (especially *Pythium* spp.) was consistently low^46^. The involvement of a species included in the *Phytophthora* sp. (clade 7, *Phytophthora sojae*), has already been reported in the literature on *Actinidia* spp. in sites affected by KVDS in Italy and Turkey^4,27^. There is also a record of the association of *Phytophthora. megasperma* var. *sojae* with kiwifruit decline in France in 1991^38^. Additionally, *Globisporangium intermedium* was reported as the main microorganism isolated from symptomatic plants of *A. deliciosa*, but the isolates were not able to incite severe symptoms of KVDS^27^. At any rate, this is in agreement with our results: a high presence of *Pythium* clade F (*Globisporangium intermedium*) counteracts the presence of *Phytophthora* sp. (clade 7), as it was especially found in the root endosphere of resistant genotypes. *Phytopythium vexans*, previously reported for its pathogenicity in kiwifruit^5,47^, has also been found in our study. However, the results presented here only partially support a prominent role for this species in KVDS, as it was found in a similar amount in the endosphere of all investigated genotypes. It may be speculated that KVDS, after having been triggered by a *Phytophthora* sp. belonging to clade 7, the most relevant oomycete implicated (according to the results presented here and elsewhere^4^), can be successively worsened by the proliferation of other opportunistic pathogens, such as *Phytopythium vexans*.

## Conclusions and remarks

To the best of our knowledge, this is the first report investigating the differential behaviour of *Actinidia* species towards soil-borne diseases. We have demonstrated that the genotypes studied had a significant impact on the survival rate of KVDS in connection with shaping the oomycete pattern. The results presented address the KVDS management strategies for the use of *A. deliciosa* or *A. chinensis* cv. grafted on *A. macrosperma* genotypes. Grafted plants need to be evaluated in the mid to long term to assess the productivity (yield) and qualitative parameters of fruits and the absence of graft incompatibility. According to the most recent phylogeny of *Actinidiaceae*, based on noncoding chloroplast DNA sequences, Tang et al., in 2019^48^ found that *A. deliciosa* belongs to a clade quite distant from *A. macrosperma*, and *A. arguta*. From a genetic point of view, the former could be considered a highly susceptible genotype, and the latter resistant/tolerant to KVDS. At any rate, *A. macrosperma* seems to tolerate the agronomic-environmental conditions in which KVDS arises very well, more than *A. arguta* under an agronomical standpoint^29^. These results should contribute to a better understanding of the influence of the oomycetes on the epidemiology of KVDS and aid in disease management and breeding programs aimed at selecting resistant/tolerant genotypes with optimal agronomical features and grafting affinity.

## Materials and methods

### Plant material

Plants used in the experiments are cuttings obtained from mother plants belonging to the *Actinidia* Germplasm repository of the University of Udine. Mother plants were collected from seeds or buds from authorized national and international repositories. They are not endangered or at risk of extinction species. All methods involving plants were carried out in accordance with relevant guidelines in the method section. The genotypes evaluated in this study were *A. macrosperma*, accession numbers 176 (♂) and 183 (♂), *A. arguta* cv. Miss Green (♀) and *A. polygama* (♂). *A. chinensis* var*. deliciosa* cv. Hayward (♀) was used as a KVDS sensitive control.

### Experimental setup

The experiment was conducted under controlled conditions in a glasshouse at the University of Udine (Italy). The plants were cultivated in 4L pots using KVDS promoting soil derived from an equal mixture of soils collected from four commercial orchards in the Friuli Venezia Giulia region (NE Italy) at a depth of 5 to 20 cm near symptomatic plants. The explanation for KVDS promotion in soils is based on the premise that these soils have already undergone testing for the presence of soil-borne pathogens associated with KVDS. However, numerous trials using susceptible genotypes have revealed that, unlike in other soils, only these specific soil types result in dysbiosis^4,29^. The sites were located at the following coordinates: site-1 (45° 59′ 55.4 N, 12° 46′ 06.6 E), site-2 (46° 00′ 31.6 N, 12° 40′ 56.3 E), site-3 (45° 50′ 43.6 N, 13° 00′ 11.5 E), and site-4 (45° 54′ 04.2 N, 12°56′45.4 E). These sites were chosen based on a previous study that found them to be KVDS-promoting^1^. Genotypes being tested were obtained in the spring of 2020 from cuttings rooted in sterile Agriperlite and were then transplanted into turf soil. They were maintained under controlled conditions throughout the 2020 growing season. In the late autumn of 2020, the plants were uprooted from the pots, the potting mix was removed, and the roots were washed in 1% sodium hypochlorite for 10 min and then rinsed in sterile water. Subsequently, the plants were potted in KVDS promoting soil. In May 2021, waterlogging conditions were replicated three times as described in literature^3^. As soon as the symptoms reached the maximum expression (more than 50% of symptomatic plants), they were evaluated, and samples of soil and roots were taken, stored, and further analysed for both the rhizosphere and endosphere compartments. We adopted 8 plants (replicates) per genotype, resulting in 8 replicates for each compartment (rhizosphere and endosphere) in all analyses performed. Genotypes are fully described in the plant material section. Figure S1 provides a summary of the workflow.

### Assessment of KVDS root symptoms and root volume

For each test-plant, the percentage of roots with necrosis or rat-tail appearance was visually assessed. The root systems were then photographed with a digital camera. Photos were processed with ImageJ (National Institutes of Health, USA)^49^ in order to confirm and refine the visual inspection data. The volume (mL) of each below-ground part was recorded by dipping the whole sample into a graduated beaker partially filled with water and measuring the pre- and -post immersion difference in water volume^37^.

### Sample collection and separation of root rhizosphere and endosphere

The roots of 8 plants per genotype were up-taken (June 2021, after symptoms appearance) and processed to separate the rhizosphere from root endosphere following the protocol of Simmons et al., 2018^50^, with some modifications. After uprooting, the samples were kept at 4 °C and processed quickly. Roots were placed into 50 ml Falcon tubes containing 30 ml of epiphyte removal buffer at pH 6.5 (6.75 g of KH_2_PO_4_, 8.75 g of K_2_HPO_4_, and 1 mL of Triton X-100, in 1 L of sterile water). The samples were sonicated at 600 Hz with a cycle of 30 s sonication and 30 without sonication for 10 min at 4 °C. The root tissues were then transferred into 30 ml of chilled (4 °C) sterile water. The tubes containing buffer and rhizosphere were centrifuged at 4000 × g for 10 min at 4 °C to let the rhizosphere deposit at the bottom of the tube. The supernatant was removed, and the rhizosphere fraction (0.2–1.0 g) was transferred into a 2 ml tube, centrifuged at 6000 g for 2 min to remove excess epiphytic buffer before immersion in liquid nitrogen and storage at − 20 °C^4^. The roots in sterilized distilled water were vortexed until all residual soil particles were removed from the surface, and 5–10 washes were necessary for complete cleaning of the roots. A total of 1 g of root tissue was processed for each plant, representing the endosphere compartment.

### DNA preparation

Total genomic DNA from 200 mg of rhizosphere of each individual sample was obtained using the Quick-DNA Fecal/Soil Microbe Miniprep kit (Zymo Research Corp.), following the manufacturer’s recommendations. Total genomic DNA from root endosphere was obtained, after freeze drying and grinding the material by using the TissueLyser II (QIAGEN) tool, then the extraction was carried out by using Total Plant DNA Spectrum kit (Merck), following the manufacturer’s recommendations. Concentration and quality of the DNA samples were verified using the NanoDrop ND-1000 spectrophotometer (ThermoFisher Scientific, Inc.). Successively DNA samples were diluted to 25 ng/μl.

### Oomycetes-specific ITS amplicon library preparation and sequencing

PCR was used to selectively amplify the ITS region of the oomycetes with the primer pair ITS3Oo/ITS4ngs^51^. Primers were deemed the most suitable for the metabarcoding analysis due to their sensitivity and ability to reduce co-amplification of vascular plant DNA. PCR conditions were modified to allow the insertion of Illumina adaptors in the primer sequences, thus circumventing the use of nested-PCR^4^, in order to gather the most precise quantitative information. The PCR mixture contained the following reagents: 12.5 μl of KAPA HiFi Hot Start Ready Mix (2 ×), 0.375 μl of each primer 20 μM and approximately 50 ng of genomic DNA, water to a final volume of 25 μl. The following thermal cycling scheme was adopted: initial denaturation at 95 °C for 3 min, 25 cycles of: 20 s at 98 °C, 15 s at 58 °C, and 15 s at 72 °C, final extension at 72 °C for 3 min. The success of the PCRs was confirmed by gel electrophoresis (2% agarose gel in 1 × TBE buffer at 120 V for 120 min). The same mixture and PCR conditions as described above were used for 15 additional cycles, with primers modified to contain the MiSeq adapters and 2 µl of each amplicon as a target. The resulting PCR products were sent to the BMR Genomics S.r.l. (Padova, Italy) for purification and sequencing on Illumina MiSeq platform using a 2 × 300 bp paired end protocol. In total, 107 samples were sequenced, including samples taken before transplanting the plants into KVDS promoting soils.

### Bioinformatics and statistical analysis

The Illumina output was received as demultiplexed FASTQ files. QIIME2 (Quantitative Insights Into Microbial Ecology part 2) software package was used for subsequent bioinformatic analysis^52^. The data (only forward reads) were processes following QIIME2 pipeline (i.e., imported as an artefact, trimmed to remove the primer sequences, filtered for quality and chimeras, and denoised using the DADA2^53^ plugin). The sequences of the 185 most frequent features were extracted and submitted to Blast analysis against the ITS_eukaryote_sequence database downloaded from NCBI in January 2023. Entries not related to the oomycetes were discarded (7% of total ASVs). The remaining ASVs were pairwise aligned with the sequence dataset used by Robideau and colleagues^35^ and assigned to the clusters identified by these authors based on percent identities. An identity score of > 99% was considered positive identification. ASVs that were > 99% similar among themselves were regarded as variants and assigned to a single OTU.

The Kruskal–Wallis test was used to assess differences in alpha-diversity between compartments and groups, with results considered significant at *α* < 0.05. The Shannon entropy, which considers both species richness and evenness, was applied.

To analyse root volume, symptoms, and the OTUs in the different samples, Shapiro–Wilk normality tests was conducted for all analyses. All tests were concluded to fit with a normal distribution and allow for statistical comparison using a parametric test. One‐way analysis of variance (ANOVA) was performed by using “R” free software (version 4.3.0 2020‐10‐10). Statistical analysis to determine significant differences between treatment means was carried out using a protected post-hoc Tukey’s honest significant difference (HSD) test (*α* < 0.05). The R library ‘multicomp letters’, which give back only the values being differently according to the threshold set up, was used to assigned different letters to significant different values. For all boxplots, the package “corrplot", plus library ‘ggplot2’ was adopted. Finally, the Pearson correlation index was used to examine the relationship between the most abundant OTUs [*Phytophthora* sp. (clade 7) and *Pythium* clade F (*Globisporangium intermedium*)] and the observed symptoms (% of necrosis on the radical system).

### Supplementary Information

Supplementary Figures.

## Data Availability

Data are available in NCBI—Sequence Read Archive (SRA), under the project code: PRJNA981691.
