# Supplementary material for: Genetic diversity of Actinidia spp. shapes the oomycete pattern associated with Kiwifruit Vine Decline Syndrome (KVDS)
Source: Sci Rep. 2023 Sep 30;13:16449. doi: 10.1038/s41598-023-43754-y (PMC10542793; doi:10.1038/s41598-023-43754-y)
Supplement: Supplementary file 1 — Supplementary Figures. [file 41598_2023_43754_MOESM1_ESM.pdf]

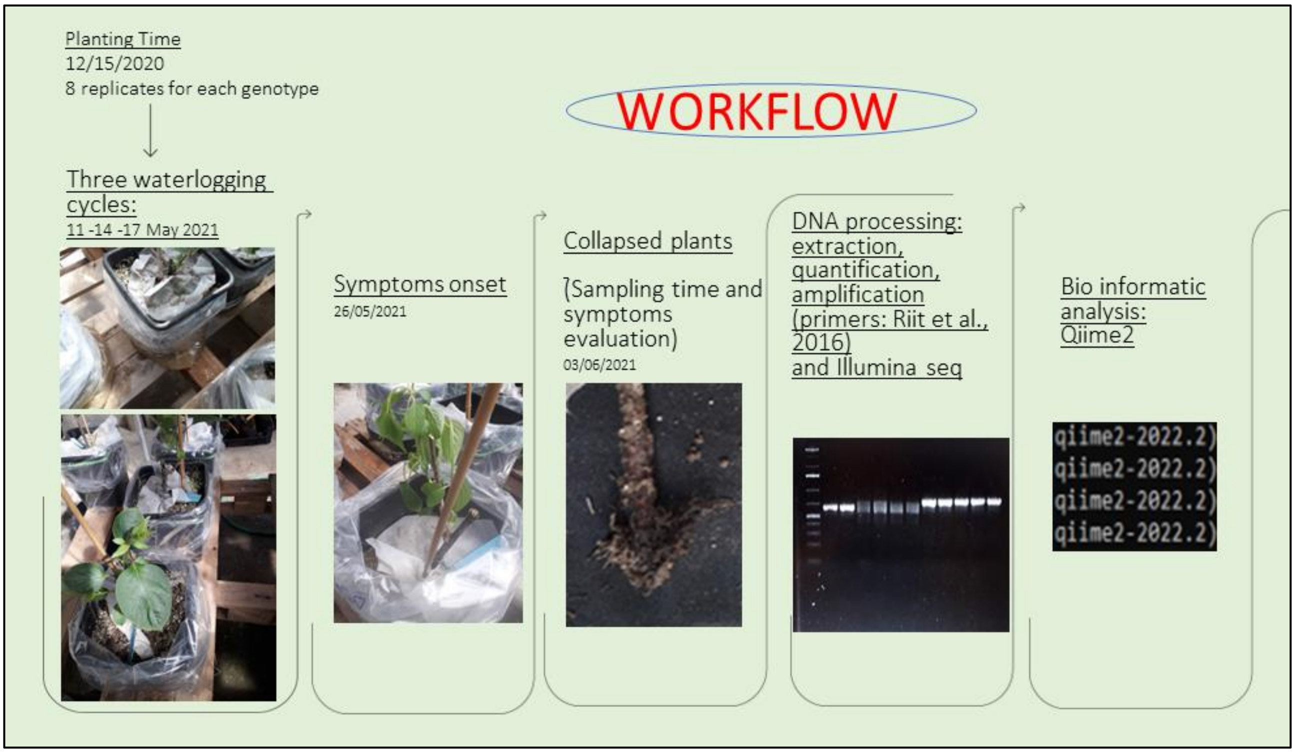

Figure S1. Workflow of the undertaken study. The picture shows the whole development starting from flooding conditions till the bioinformatic analyses.

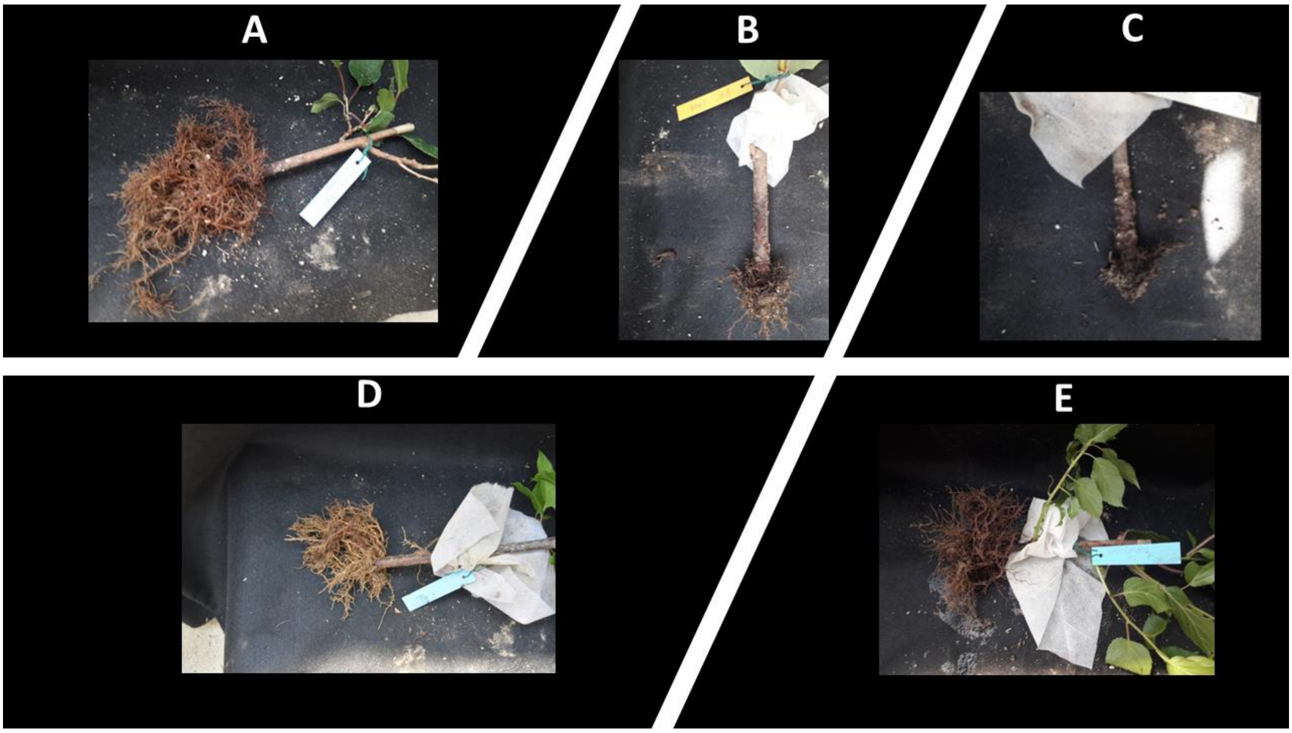

Figure S2. Examples of root system at the time of maximum expression of symptoms. A: *A. macrosperma*-Ma176, B: *A. polygama*-Pol, C: Hw- *A. deliciosa* cv. Hayward, D: MG- *A. arguta* cv. Miss Green, E: *A. macrosperma*-Ma183.

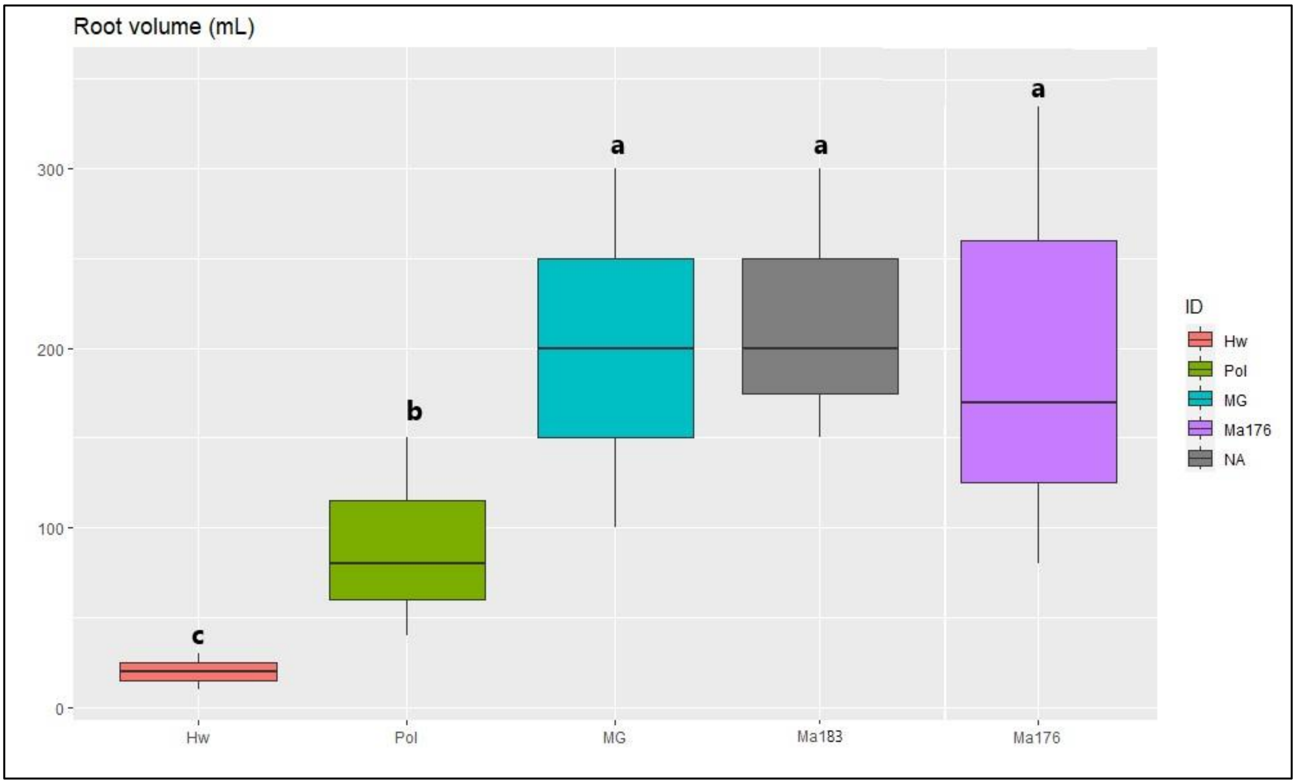

Figure S3. Root volume at the time of maximum symptom expression. Data are the mean of 8 replicates per genotype. Statistical analysis (ANOVA) was carried out using the Tukey HSD test using R version 4.3.0. Values assigned with different letters are significantly different at $\alpha<0.05$. Hw: *A. deliciosa* cv. Hayward, Pol: *A. polygama*, MG: *A. arguta* cv. Miss Green., Ma183 and Ma176: *A. macrosperma*.

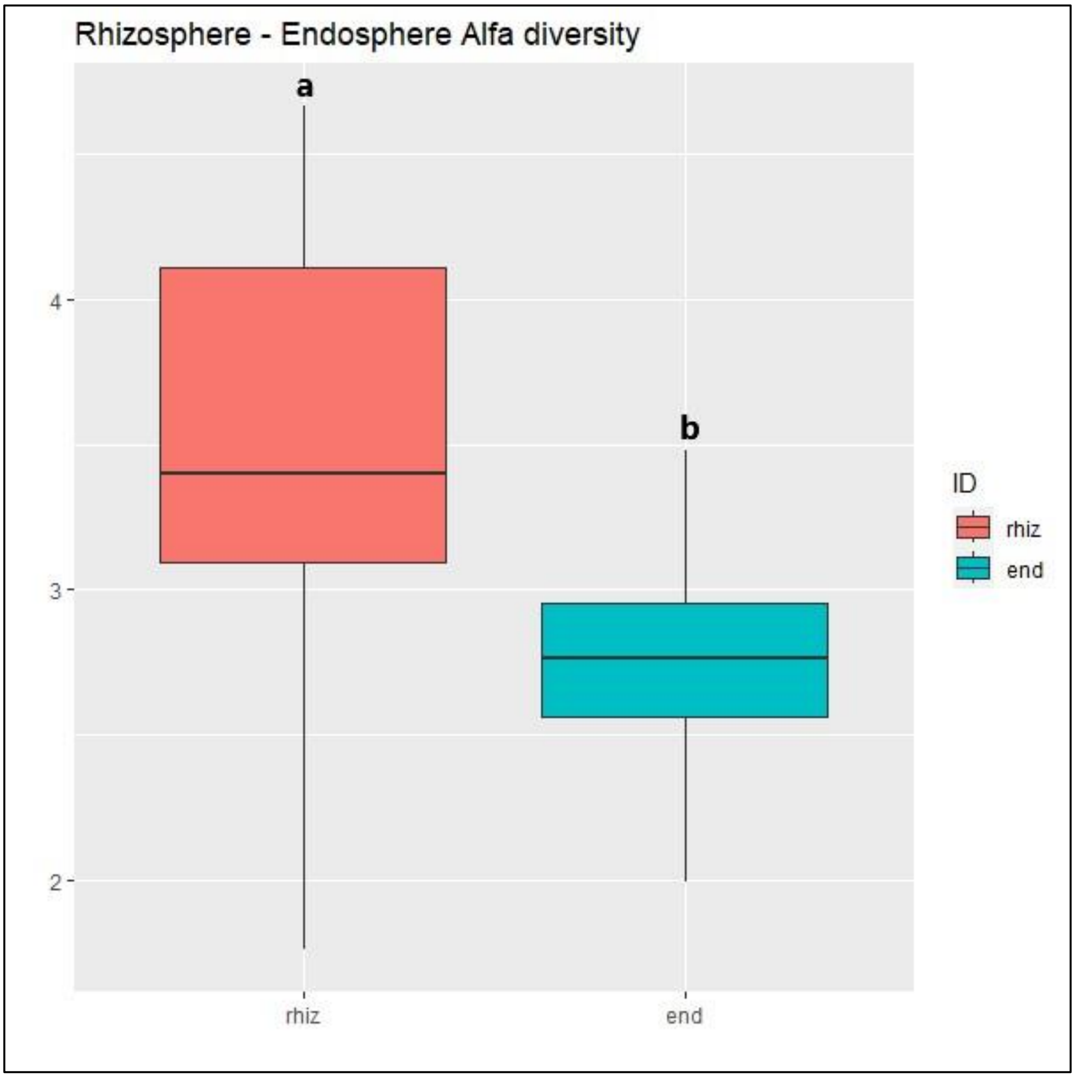

Figure S4. Statistical analysis of alpha-diversity comparing the diversity of the oomycetes in root rhizosphere and endosphere at the moments of maximum symptoms expression. Data are the mean of 8 replicates per genotype. Statistical analysis (ANOVA) was carried out using the Tukey HSD test using R version 4.3.0. Values assigned with different letters are significantly different at $\alpha<0.05$.

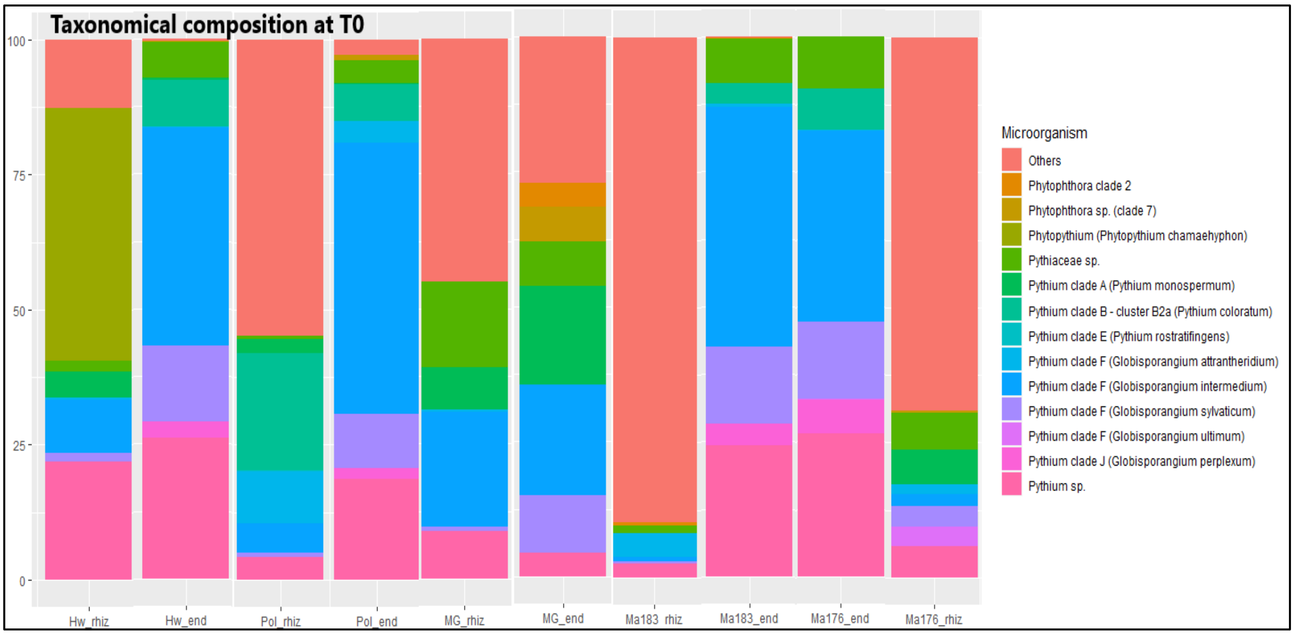

Figure S5. Taxonomic composition of the oomycetes found in the rhizosphere and endosphere of different *Actinidia* spp. before transplanting in KVDS promoting soil. Hw: *A. deliciosa* cv. Hayward, Pol: *A. polygama*, MG: *A. arguta* cv. Miss Green, Ma176 and Ma183: *A. macrosperma*. rhiz: rhizosphere, end: endosphere. Data are expressed as relative abundance (%) given by the number of reads for each microorganism on the total number of reads.

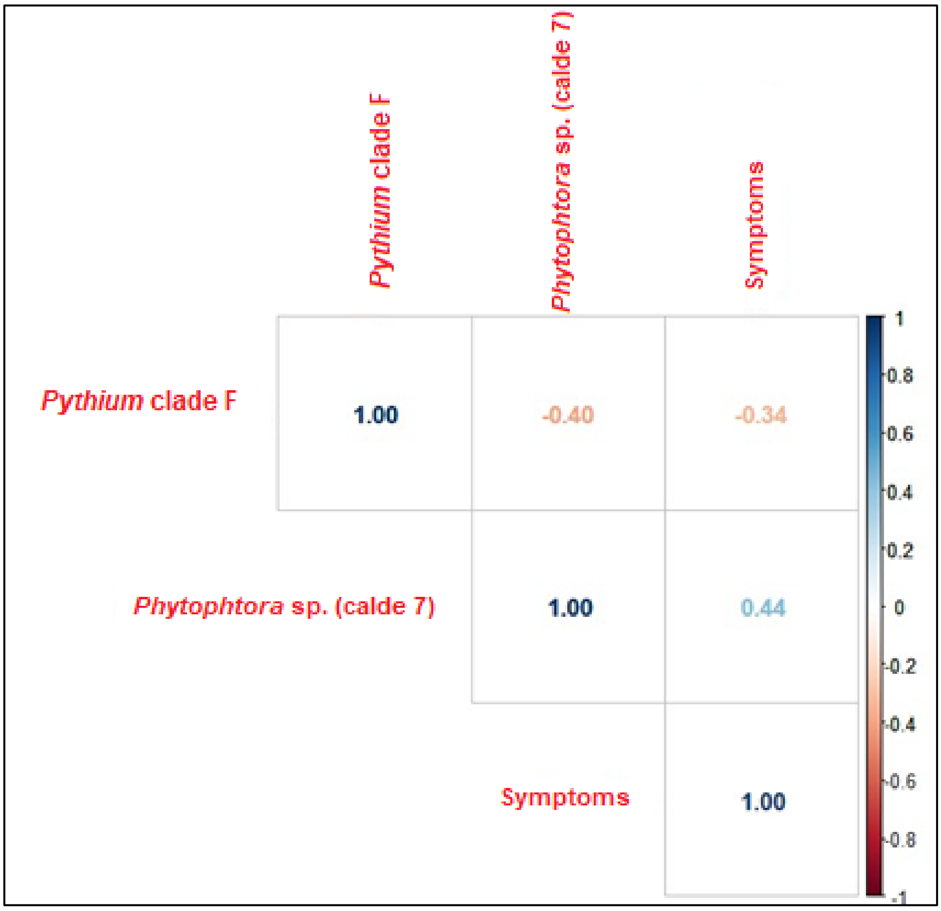

Figure S6. Pearson correlation index plotted with R version 4.3.0. Data refer the root endosphere of each plant group, considering only *Phytophthora* sp. (clade 7), *Pythium* clade F *(Globisporangium intermedium*) and symptoms.

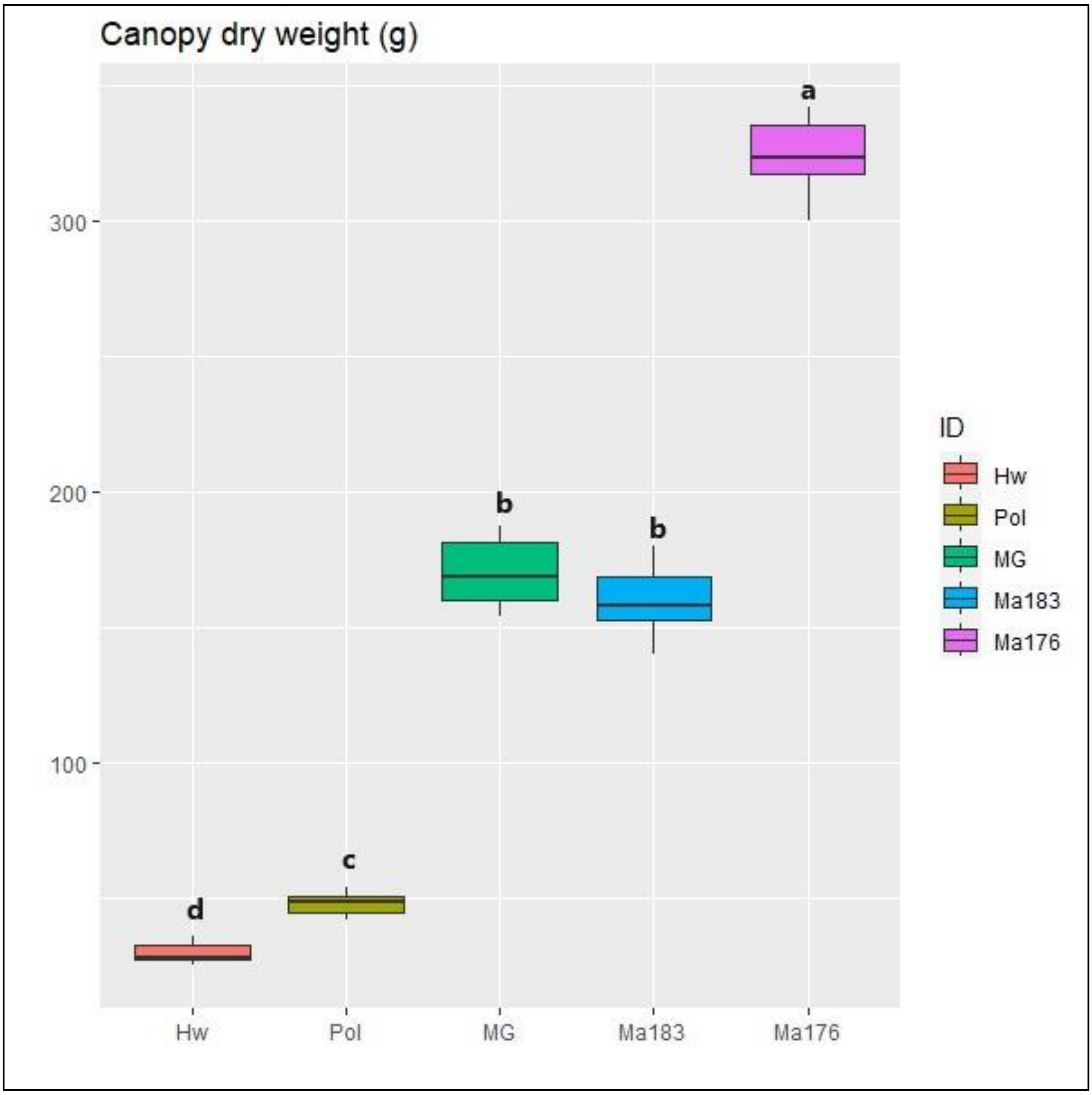

Figure S7. Canopy dry weight (g) recorded after the vegetative season. Data are the mean of 8 replicates per genotype. Statistical analysis (ANOVA) was carried out using the Tukey HSD test using R version 4.3.0. Values assigned with different letters are significantly different at α<0.05. Hw: *A. deliciosa* cv. Hayward, Pol: *A. polygama*, MG: *A. arguta* cv. Miss Green., Ma176 and Ma183: *A. macrosperma*.
